# Intracellular remodeling associated with endoplasmic reticulum stress modifies biomechanical compliance of bladder cells

**DOI:** 10.1186/s12964-023-01295-x

**Published:** 2023-10-30

**Authors:** Livia Gruber, Maximilian Jobst, Endre Kiss, Martina Karasová, Bernhard Englinger, Walter Berger, Giorgia Del Favero

**Affiliations:** 1 Department of Food Chemistry and Toxicology, University of Vienna Faculty of Chemistry, Währinger Str. 38-40, Vienna, 1090 Austria; 2 Core Facility Multimodal Imaging, University of Vienna Faculty of Chemistry, Währinger Str. 38-40, Vienna, 1090 Austria; 3 University of Vienna, Vienna Doctoral School in Chemistry (DoSChem), Währinger Str. 42, Vienna, 1090 Austria; 4 Department of Urology, Comprehensive Cancer Center, Medical University of Vienna, Vienna, 1090 Austria; 5 Center for Cancer Research and Comprehensive Cancer Center, Medical University Vienna, Vienna, 1090 Austria

**Keywords:** Brefeldin A, Thapsigargin, Cell stiffness, Cytoskeleton, Endoplasmic reticulum stress, Atomic force microscopy

## Abstract

Bladder cells face a challenging biophysical environment: mechanical cues originating from urine flow and regular contraction to enable the filling voiding of the organ. To ensure functional adaption, bladder cells rely on high biomechanical compliance, nevertheless aging or chronic pathological conditions can modify this plasticity. Obviously the cytoskeletal network plays an essential role, however the contribution of other, closely entangled, intracellular organelles is currently underappreciated. The endoplasmic reticulum (ER) lies at a crucial crossroads, connected to both nucleus and cytoskeleton. Yet, its role in the maintenance of cell mechanical stability is less investigated. To start exploring these aspects, T24 bladder cancer cells were treated with the ER stress inducers brefeldin A (10-40nM BFA, 24 h) and thapsigargin (0.1-100nM TG, 24 h). Without impairment of cell motility and viability, BFA and TG triggered a significant subcellular redistribution of the ER; this was associated with a rearrangement of actin cytoskeleton. Additional inhibition of actin polymerization with cytochalasin D (100nM CytD) contributed to the spread of the ER toward cell periphery, and was accompanied by an increase of cellular stiffness (Young´s modulus) in the cytoplasmic compartment. Shrinking of the ER toward the nucleus (100nM TG, 2 h) was related to an increased stiffness in the nuclear and perinuclear areas. A similar short-term response profile was observed also in normal human primary bladder fibroblasts. In sum, the ER and its subcellular rearrangement seem to contribute to the mechanical properties of bladder cells opening new perspectives in the study of the related stress signaling cascades.

Video Abstract

**Supplementary Information:**

The online version contains supplementary material available at 10.1186/s12964-023-01295-x.

## Introduction

The endoplasmic reticulum (ER) is often described as the biggest organelle of a eukaryotic cell [1]. The ER hosts crucial processes like protein and lipid biosynthesis, as well as playing an essential role in intracellular calcium ([Ca^2+^]_i_) management [2, 3]. Hence, in order to comply with the physical constraints of the cytoplasmic compartment, the membrane of the ER is neatly folded and organized. This achieves a compact spatial distribution and at the same time ensures cell structural stability. From the morphological perspective, it is possible to identify different structures in the ER; either flat reservoirs (sheets) or elongated cylinders (tubules) [4] which harbor specific structural proteins, as well as defined molecular functions. As the ER mostly “embraces” the cell nucleus, the two organelles are in close contact with each other, and according to this view, the perinuclear region of the ER prolongates the nuclear envelope [5]. From this point, the ER membrane expands progressively toward the cell periphery [6, 7] with distal structures. Considering the conspicuous mass of the ER within the cell, it is clear that structural adaption has to occur not only for the rearrangement of the organelle itself, but also to accompany cell motility [8]. Hence, the ER is tightly linked to cytoskeletal proteins, as it was previously described that filamin A is essential to connect the cell cytoskeleton to the ER [9] and ensure respective mutual adaption. On a similar note, previous work demonstrated that ER movement along the microtubules occurs on the filaments that have been stabilized post-translationally via acetylation [10]. It has also been shown that actin depolymerization via cytochalasin D (CytD) determines a spread of the ER network in the cytoplasmic compartment [11]. Indeed, selective pharmacological manipulation of cytoskeletal elements, such as actin or microtubules, changes the appearance of the ER with the adaption of the ER sheet/tubules proportion [12]. Moreover, rather recently the ER stress sensing protein and unfolded protein response (UPR) mediator IRE1 has been described to govern cytoskeleton remodeling and cell migration via the interaction with filamin A, independent of its role in the UPR [13]. In spite of the clear connection between the ER and the cytoskeleton, comparatively little is known on the capacity of the organelle to contribute to cell biomechanical compliance [14]. Along this line, it was previously suggested that intracellular organelles, such as the mitochondria could contribute to generate physical signals [15]; supporting not exclusively metabolic needs, but also playing a role in the maintenance of cell structure. More acknowledged is the role of the cell nucleus in this respect. Hence, it is known that the nucleus has to adapt to enable cell migration and motility [16, 17]. In this regard, mutations in the nucleoskeletal proteins such as the lamins are associated with altered nuclear stiffness which possibly contributes to loss of physical adaptive capacity from single cells to entire tissues [18–22]. Similarly, loss of lamin A/C was related to increased metastatic motility in breast cancer cells, possibly supporting enhanced deformability of the nucleus and favoring in this way invasion of other tissues and consequently tumor aggressiveness [23].

On these molecular premises, it appears plausible that intracellular organelles might contribute substantially to cell biomechanical compliance. This infers for a structural role in addition to the known biochemical functions of the individual entities. Taking this as a starting point, we postulated a contribution for the ER in supporting cell shape and integrity. Hence, it is possible to envision that ER stress responses could relate to the rearrangement of the organelle, but also participate to alter cell structure and biomechanical compliance. In order to start exploring this hypothesis, T24 bladder cancer cells were incubated with known ER stress triggers such as brefeldin A (BFA) [24], and thapsigargin (TG) [25], which are able to modify the structure and the function of the ER-cytoskeletal network via distinct mechanisms. TG is a specific inhibitor of the sarco/endoplasmic reticulum Ca^2+^-ATPase (SERCA) [26]. Additionally, it triggers ectopic calcium influx that has been described to lead to actin depolymerization via cofilin-1 and mTOR-RhoA pathways [27]. BFA on the other hand, is a widely used inhibitor of ER-Golgi transport that blocks the activity of guanine-nucleotide exchange factors (GEF) to activate ADP-ribosylation factors (ARF) [28] including ARF6. This in turn plays an important role in actin cytoskeleton rearrangements at the plasma membrane [29]. With this experimental layout, the impact of the treatments on the morphology of the ER and on the actin cytoskeleton was tested. Once the long term (24 h) and short term (2 h) morphometric adaption capacity of the ER was defined, cell biomechanical compliance was described via atomic force microscopy (AFM), to enable a mapping of cell stiffness (Young´s modulus) and its subcellular spatial distribution [30, 31]. In sum, we generated a toolbox that supports the description of the morphological changes of the ER and correlated it to the effects on cell biomechanical response capacity.

## Materials and methods

### Chemicals and reagents

Brefeldin A (BFA, REF: 3660755, 10-40nM [24, 32]), thapsigargin (TG, REF: 3557977, 0.1-100nM [33, 34]), wortmannin (WORT, REF: W3144, 1µM [35, 36]) and cytochalasin D (CytD, REF: C2618, 100nM [37, 38]) were purchased from Sigma-Aldrich (Burlington, Massachusetts, United States). Stock solutions of the compounds were prepared in dimethyl sulfoxide (DMSO; Carl Roth, Karlsruhe, Germany) to enable a 1:1000 dilution for each treatment. Solvent controls were matched to represent the same DMSO content as the treatments. PBS-A was prepared by dissolving 0.4g KH_2_PO_4_, 0.4g KCl, 16g NaCl and 5.5g Na_2_HPO_4_. 2H_2_O in 1l autoclaved water and adjusting the pH to 7.2.

### Cell culture

The urinary bladder carcinoma cell line T24 (ATCC® HTB4™ USA) was purchased from the American type culture collection (ATCC; Virginia, USA). Cells were cultivated according to the specification of the supplier in McCoy´s 5a medium (Gibco, Netherlands) supplemented with 10% (v/v) heat-inactivated fetal bovine serum (FBS) and 1% (v/v) Penicillin/Streptomycin in TC-Flasks T25/T75 (Sarstedt, Nümbrecht, Germany). Normal, human, primary bladder fibroblasts (PCS-420–013™) were purchased from the American type culture collection (ATCC; Virginia, USA). Cells were cultivated in Fibroblast Basal Medium (ATCC, PCS-201–030) supplemented with Fibroblast Growth Kit-Low Serum (ATCC PCS-201–041), containing final concentrations of 5ng/ml rhFGF, 7.5mM L-glutamine, 50µg/ml ascorbic acid, 1µg/ml hydrocortisone hemisuccinate, 5µg/ml rh insulin, and 2% FBS. For cultivation and incubations, humidified incubators were used at 37 °C and 5% CO_2_ if not otherwise specified. Cells were regularly passaged when reaching a confluency of 80 – 90% (3 times a week).

### Cell viability (WST-1 assay)

Cell viability was assessed using the WST-1 assay as previously described [39]. Briefly, T24 cells were seeded in 96-well plates and incubated for 24 h. Next, the cells were treated with different concentrations of BFA and TG. The cells were incubated for a further 24 h. Then the cells were washed with phenol red- and serum-free Dulbecco’s modified Eagle medium (DMEM; Gibco, Netherlands), followed by the addition of the WST-1 reagent (dil. 1:10 in phenol red- and serum-free DMEM, Roche, 116448807001 St. Louis, USA). After 30 min of incubation, the absorbance was measured at 440 nm and as a reference at 650 nm using a multi-detector microplate reader Synergy H1 Hybrid (BioTek, Bad Friedrichshall, Germany). At least 3 independent biological replicates were performed, with five technical replicates for each condition.

### Migration assay

The migration assay was performed as previously described [40], T24 cells were seeded in 35 mm 6 well plates (REF: 83.3920.005, Sarstedt, Nümbrecht, Germany) in 2 ml cell culture medium. After 48 h, at the point of almost full confluence, the scratch was performed using a 200 µL pipette tip. The cells were incubated for 24 h with the respective treatments in 2 ml culture medium. The first set (t = 0 h) of phase contrast pictures was taken on a Lionheart FX automated microscope using the GEN5 microplate and Imager software version 3.05. for imaging and quantification (BioTek Instruments Inc., Vermont, USA). After 24 h of incubation, a second image set at the same coordinates was taken. 4 independent biological replicates were performed, for each 3 optical fields were evaluated, resulting in a total 12 optical fields per condition. Quantification was performed using ImageJ version 1.53a, results are given as area healed [µm^2^] (Wound area [t = 0 h] - Wound area [t = 24 h]).

### Live cell imaging and microscopy

For the evaluation of ER morphology, live cell imaging experiments were performed as previously described [11, 41, 42] with minor modifications. Briefly, cells were seeded 24 h before treatment in Ibidi IbiTreat μ-slide 8 well slides (REF: 80.826, Ibidi GmbH, Gräfelfing, Germany). After treatment, cells were washed two times with prewarmed (37°C) live cell imaging solution (REF: A14291DJ, Thermo Fisher Scientific, Invitrogen) and subsequently incubated with the ER staining reagent Green Detection Reagent (REF: ab139481, dil. 1:2000, Abcam) and CellMaskTM Deep Red Plasma membrane stain (REF: C10046, dil. 1:2000 Thermo Fisher Scientific) for 15 min in LCIS at 37°C with 5% CO_2_ supply. After a subsequent washing step, the stained cells were kept in LCIS and imaged using a Zeiss LSM710 laser scanning confocal microscope (ELYRA PS.1 system) equipped with a 63X/1.2 water immersion objective. For image analysis, ImageJ (Version 2.9.0) was used. Regions of interest (ROIs) were drawn in 6 cells per optical field. Within the selections, the mean of the ER fluorescence signal intensity was measured in relative fluorescence units. To determine the area of the ER within the cell, the percentage of pixels corresponding to the ER signal in selected cells was measured. Each dataset resulted from the analysis of a minimum of 3 independent cell preparations (biological replicates). For experiments monitoring live ER rearrangement, kinetic data were generated (0-0.5-1-2 h). For this, the Lionheart FX automated microscope with the GEN5 microplate and Imager software version 3.05 was used for imaging and quantification (BioTek Instruments Inc., Vermont, USA). Here the area of the ER was evaluated taking time 0 (before TG application) as a starting point. Average values for optical field were determined and are expressed as mean of n ≥ 16 optical fields from n = 3 independent cell preparations.

### Immunofluorescence staining and microscopy

Immunofluorescence experiments were performed as previously described [11, 42]. Briefly, cells were seeded 24 h before treatment in Ibidi IbiTreat μ-slide 8 well slides (REF: 80.826, Ibidi GmbH, Gräfelfing, Germany). After treatment, cells were fixed with 3.5% formaldehyde (FA) in DPBS for 15 min at RT. Afterwards, cells were permeabilized with 0.2% Triton X-100 in phosphate-buffered saline (PBS-A) for 15 min at RT followed by blocking with 2% donkey serum (Sigma Aldrich, A9663) for 1 h at RT. Cells were then incubated with primary antibodies (dil. 1:500, IRE-1 ab370073 Abcam, rabbit polyclonal; dil. 1:1000, CHOP MA1-250, Invitrogen, mouse monoclonal) for 2 h at RT. After the incubation with the primary antibody, the cells were washed 3 times with 0.05% Triton X-100, 10 min each. After 2 additional washing steps with PBS-A for 5 min each, Alexa FluorTM 568 donkey anti-rabbit IgG (H + L) (dil. 1:1000, Molecular Probes-Life Technologies) or Alexa FluorTM 647 donkey anti-mouse IgG (H + L) (dil. 1:1000, Invitrogen) as secondary antibody was applied together with Phalloidin (dil. 1:500, Oregon Green 488 Phalloidin, Invitrogen) for actin filament staining. The cells were incubated at RT in the dark for 1.5 h. After 3 washing steps with 0.05% Triton X-100 and 2 washing steps with PBS-A, cells were incubated with 3.5% FA for 10 min at RT as post-fixation step. After that, cells were washed once with PBS-A and FA was quenched with 100mM glycine in PBS-A. Cells were embedded in DAPI containing mounting medium (ab104139, Abcam). A Zeiss LSM710 laser scanning confocal microscope (ELYRA PS.1 system) equipped with a 63X/ 1.4 plan-apochromat oil immersion objective (Zeiss Microscopy GmbH, Germany) was used for imaging. Image analysis was performed with ImageJ. The mean fluorescence intensity of IRE1, CHOP and actin staining was measured in nuclear and cytoplasmic compartments within regions of interest (ROIs). The cell nuclei staining (DAPI) and the actin cytoskeleton staining were used as reference for the selection. Each dataset resulted from the analysis of a minimum of three independent cell preparations (biological replicates). Morphology of DAPI stained nuclei was quantified using ImageJ version 1.54f, after thresholding, nuclear masks were detected and the parameters area, circularity (4*π*(area/perimeter^2^)), aspect ratio (major axis/minor axis), solidity (area/convex area) and roundness (4*area/(π* (major axis)^2^)) were measured [43]. Average nuclear parameters per optical field were used, in total n ≥ 12 optical fields were evaluated, for 3 independent biological replicates.

Evaluation of the nuclear – cytoskeletal distances was performed in ZEN black edition software (Zeiss Microscopy GmbH, Germany) from cross-sectional intensity profiles, conducted in the direction of longer axis of the nucleus/cell. Using the nuclear borders as reference and actin intensity peaks of 50 r.f.u., values on both sides of the nuclei were averaged to provide individual cell values, to compensate eventual asymmetric distribution of the ER. Data results from the quantification of n ≥ 35 cells, for 3 independent cell preparations (biological replicates).

### Atomic Force Microscopy (AFM)

An AFM (JPK NanoWizard® 4 XP, Bruker, Germany) coupled with an inverted Olympus IX73 optical microscope was used to investigate the mechanical properties of living T24 cells upon treatment with BFA (20nM), TG (1-100nM), CytD (100nM), Wortmannin (1µM) as well as co-incubation of BFA and TG with CytD. Cells were seeded in tissue culture dishes (REF: 40 93040, TPP Techno Plastic Products AG, Switzerland) for 24–48 h for T24 and 48–72 h for fibroblasts to obtain cell density of 70–80%. After this period, cells were incubated with the respective treatments for 2 to 24 h. Following incubation, cells were washed once with pre-warmed LCIS and subsequently imaged in LCIS using QI™ mode and PFQNM-LC-A-CAL AFM tips (Bruker) with calibrated spring constant k ranging from 0.125 N/m to 0.144 N/m. QI™ settings used are the following: Z-length: 1000 nm; applied force 0.2 nN; speed 100 µm/s [44]. Living cells were randomly selected using the optical microscope and imaged in a 25 µm × 25 µm square area. Cells were imaged for a maximum of 60 min to ensure stable cell viability. In total, 13 to 32 cells for each experimental condition derived from at least 3 independent cell preparations (biological replicates) were imaged and force curves were processed to obtain data of the Young´s modulus. The raw data was processed using JPK NanoWizard® Data processing software. Within the images, an area of 3.1 µm × 3.1 µm was selected in the nuclear, perinuclear or cytoplasmic region. A representative force curve of the cell was chosen to set up the processing of the Young’s modulus. First, through the calculation of the average value of a section in the baseline and subsequent subtraction of the whole curve, the baseline offset in vertical deflection was removed. Then, the vertical deflection was plotted against the vertical tip position. Afterwards, the reference force height was determined at the height value at 50% of the applied setpoint force. Finally, elasticity fit was used to calculate the Young´s modulus. Here, the Hertz/Sneddon fit [45, 46] was used for a paraboloid tip shape with a 70 nm tip radius and a Poisson´s ratio value of 0.50 [47, 48]. Force curves were batch-analyzed in the selected area of the nuclear, perinuclear, and cytoplasmic regions and median values of the Young´s modulus were calculated [49].

### Statistical analysis

All data were statistically evaluated and plotted using the software OriginPro 2022 version 9.9 and 2023 version 10. Statistical analyses were performed using Student’s *t*-test (n > 33) for LCI and immunofluorescence imaging and Mann–Whitney test (n < 32) for migration and AFM data. For concentration dependent datasets, ANOVA test with Fisher LSD was used. For all statistics, p values smaller than 0.05 were considered statistically significant. At least three independent cell preparations (biological replicates) were used for all experiments.

## Results

### Effects of ER stress on cell viability and endoplasmic reticulum morphology

**Fig. 1:**
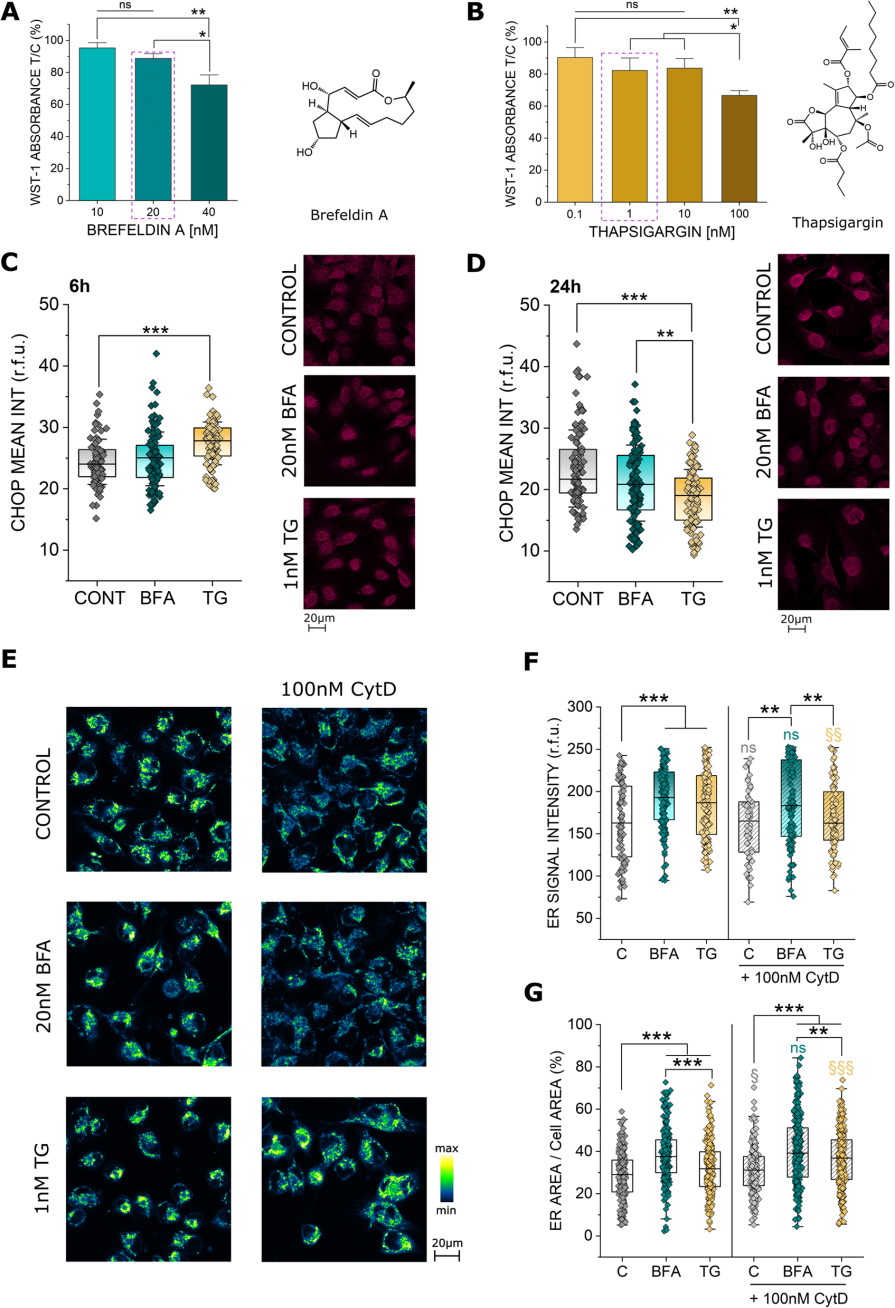
**A** Effect of brefeldin A (BFA, 10-40nM) and **B** thapsigargin (TG, 0.1-100nM) on T24 cell viability measured as metabolic activity (WST-1). Cell viability is depicted as % of the control. * indicates significant difference at the one-way ANOVA with Fisher LSD test (*: *p* ≤ 0.05; **: *p* ≤ 0.01), ns indicates no significant difference (*p* > 0.05), 5 technical, n = 3 biological replicates were performed. Quantification and representative images of CHOP staining in T24 cells treated for 6 h (**C**) and 24 h (**D**) with TG (1nM) or BFA (20nM). CHOP is depicted in magenta. At least n ≥ 87 ROIs were quantified taken from at least 3 independent cell preparations. * indicates significant differences in comparison to controls at the Student’s t-test (**: *p ≤* 0.01; ***: *p* ≤ 0.001). **E** Representative images of the ER distribution (blue to yellow), **F** quantification of the ER signal intensity (r.f.u.) and** G** quantification of the ER area per cell area (%) of T24 cells, control cells and 24 h incubation with BFA and TG and CytD. Grey: §/ns indicates differences to controls at the Student’s t-test (ns: *p* > 0.05; §: *p* ≤ 0.05). Cyan: ns indicates no significance between BFA incubations with or without Cytochalasin D at the Student’s t-test (ns: *p* > 0.05). Yellow: § indicates significance between TG incubations with or without Cytochalasin D at the Student’s t-test (§§: *p* ≤ 0.01; §§§: *p* ≤ 0.001). Black: * indicates significant differences in comparison to controls at the Student’s t-test (**: *p* ≤ 0.01; ***: *p* ≤ 0.001). Data were acquired from 3 independent cell preparations n > 70 ROIs/cells

In order to investigate a potential role for the endoplasmic reticulum in the maintenance of cell biomechanical compliance, initial concentration range finding experiments were carried out. Since it was previously described that increasing ER stress can activate an apoptotic cascade in the cell [50] and unspecific cytotoxicity would most likely per se modify cell biomechanical compliance, sub-cytotoxic concentrations of the ER stressors brefeldin A (BFA) and thapsigargin (TG) were selected for further analysis. In the WST-1 assay (Fig. 1A, B), both molecules elicited a concentration dependent effect; this manifested with a decrease of cell viability up to 80% reduction (40nM BFA and 100nM TG). Hence, 20nM BFA and 1nM TG were chosen for further experiments. To confirm the induction of ER stress in these conditions, the response of the transcription factor CHOP [33, 51] was investigated. Along this line, BFA (20nM) slightly increased CHOP signal and TG significantly enhanced the transcription factor (Fig. 1C, 1nM, 6 h). Following a kinetic of activation and degradation of transcription factors, 24 h incubation returned a mirroring signal decrease (Fig. 1D). In order to verify if the stressors also induced a structural adaptation of the ER, the morphology and subcellular distribution of the organelle were evaluated (Fig. 1E). In agreement with the described role as ER stress inducers [24, 26], both BFA and TG increased the ER signal per cell (Fig. 1F). Additionally, the ER spread increased in the intracellular compartment, as visible in the measurement of the area covered by the organelle in respect to the whole cell (Fig. 1G). Since the ER exists in close relation with the actin cytoskeleton [11, 52, 53], the behavior of the ER was observed also in presence of the actin inhibitor cytochalasin-D (CytD). CytD had no effect on the intensity of the ER signal in controls or BFA treated cells, however it significantly reduced the response elicited by TG (Fig. 1F). Furthermore, fitting previous data [11], CytD increased the spread of the ER. Even if the morphometric rearrangement triggered by BFA alone could not be further enhanced by the presence of CytD (Fig. 1G), this response could be clearly observed for controls and TG treated cells.

### Effects of ER stress on IRE1 and nuclear morphology

**Fig. 2:**
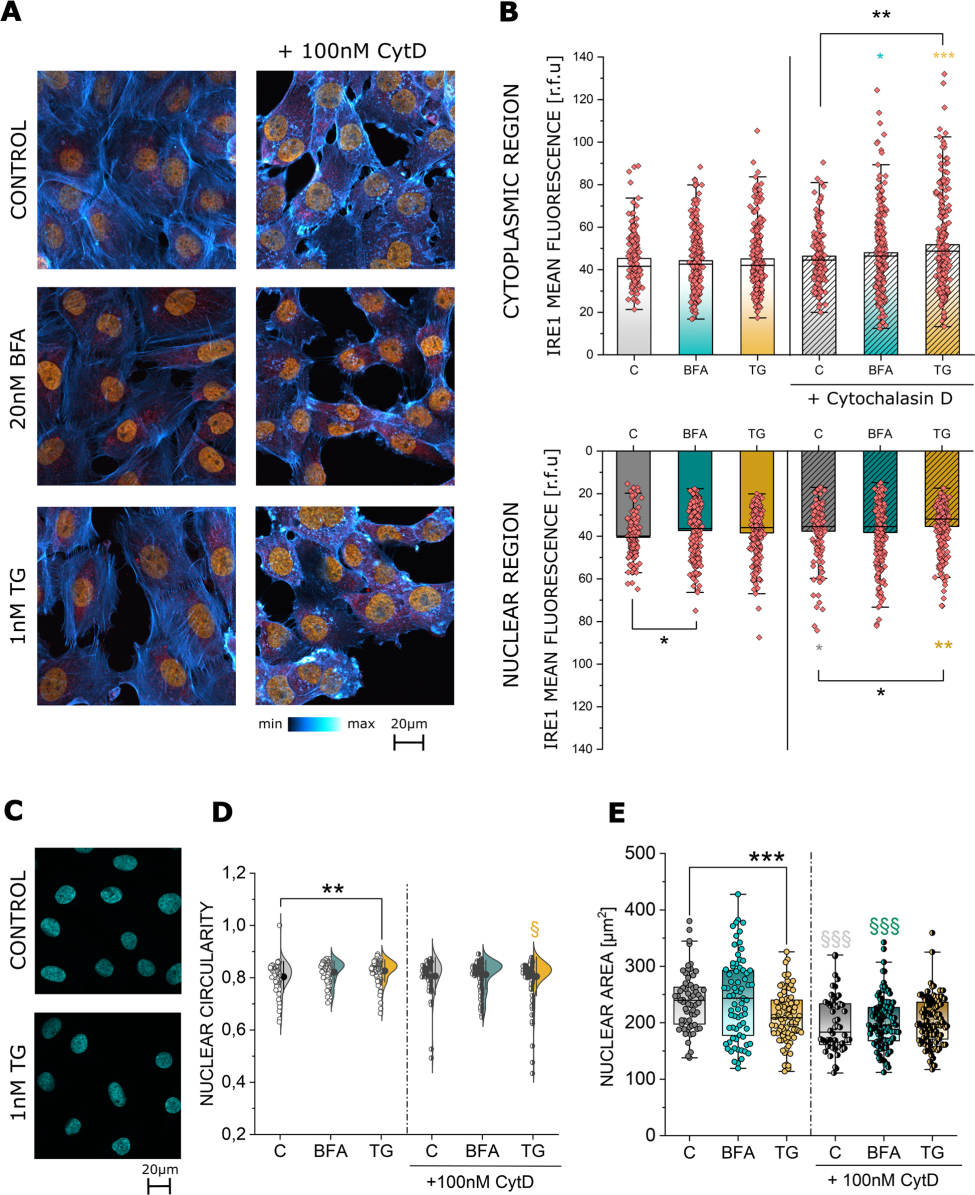
**A** Representative images of the immunofluorescence staining of IRE1 (red), actin (blue to white) and the nucleus (orange) of T24 cells after 24 h incubation in control conditions and with 20nM BFA and 1nM TG as well as after co-incubation with 100nM CytD. **B** Quantification of IRE1 signal intensity in cytoplasmic and nuclear region of T24 cells as relative fluorescent units (r.f.u.) after 24 h incubation with BFA, TG and their combination with CytD. Yellow symbols: § indicates significant difference between TG incubations with and without CytD. Cyan symbols: § indicates significant difference between BFA incubations with and without CytD (§: *p* ≤ 0.05; §§: *p* ≤ 0.01; §§§: *p* ≤ 0.001). Black symbols: * indicates significant difference between treatments (*: *p* ≤ 0.05; **: *p* ≤ 0.01). Data was acquired from 3 independent cell preparations n > 70 ROIs. **C** Representative image of DAPI (Cyan) stained nuclei of T24 cells after 24 h incubation in control conditions and TG treatment. **D** Quantification of nuclear circularity, statistical significance to control is at Student´s t-test (**: *p* ≤ 0.01) yellow § indicates difference between TG and TG in combination with CytD. Data were acquired from 3 independent cell preparations n > 50 cells. **E** Quantification of nuclear area results, statistical significance to control at Student´s t-test (***: *p* ≤ 0.01) grey and cyan §§§ indicates difference between controls/BFA and controls/BFA in combination with CytD. Data were acquired from 3 independent cell preparations n > 50 cells

To further determine if the morphological changes of the ER triggered by BFA, TG and CytD also translate into an alteration of ER function, the response of the IRE1 was explored. IRE1 is an acknowledged marker of the ER stress and the unfolded protein response (UPR) [54] as well as a regulator of the cytoskeleton during cell movement [13] (Fig. 2A). To follow the spatial distribution of the ER, IRE1 signal quantification was obtained selecting ROIs localized in the cytoplasmic and in the nuclear region of the T24 cells. While neither BFA nor TG elicited a change in the cytoplasmic signal of IRE1, the protein was significantly reduced in the nuclear region following 24 h of BFA treatment (Fig. 2B). The application of CytD with the ER stressors triggered a significant increase in the IRE1 localization in the cytoplasmic region. In the nuclear region, CytD incubation alone as well as the combination with TG was accompanied by a decrease in IRE1 signal in comparison to controls. Additionally, since the ER is in close contact with the nucleus, the morphology of the latter was quantified to observe if a rearrangement of the ER network could also correspond to a variation of the nuclear appearance (Fig. 2C-E). In this context, TG was more efficient than BFA in reshaping nuclear morphology, hence its presence significantly decreased nuclear area (Fig. 2E) while increasing its circularity (Fig. 2D). To further confirm the connection between the cyto-nucleoskeletal components, the use of CytD also triggered a reduction of the nuclear area, yet no parallel change in nuclear circularity was observed (Fig. 2D, E).

### Effects of ER stress on the actin cytoskeleton and cell migration

**Fig. 3:**
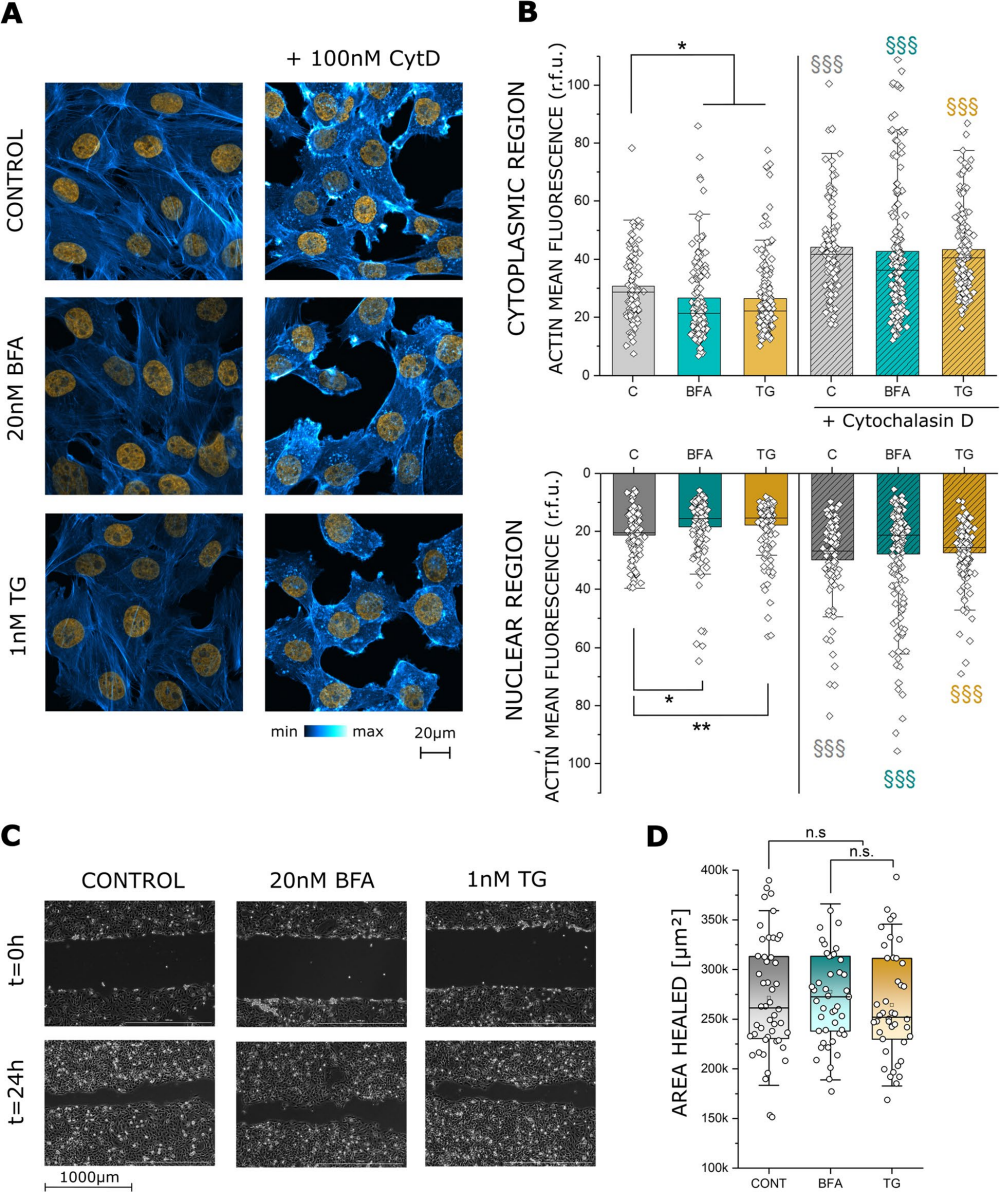
**A** Fluorescence staining of actin (blue to white) and the nucleus (orange) in T24 cells after 24 h incubation in control conditions and with 20nM BFA and 1nM TG as well as after co-incubation with 100nM CytD. **B** Quantification of actin signal intensity in cytoplasmic and nuclear region of T24 cells as relative fluorescent units (r.f.u.) after 24 h incubation in control conditions, BFA, TG and their combination with CytD. Grey symbols: § indicates significant difference between control incubations with and without CytD. Yellow symbols: § indicates significant difference between TG incubations with and without CytD. Cyan symbols: § indicates significant difference between BFA incubations with and without CytD (§§§: *p* ≤ 0.001). Black symbols: * indicates significant difference between treatments (*: *p* ≤ 0.05; **: *p* ≤ 0.01). Data were acquired from 3 independent cell preparations n > 70 ROIs. **C** Representative images of the gap-closure migration assay at t = 0 and t = 24 h. Control cells as well as 24 h incubation with 20nM BFA and 1nM TG. **D** Quantification of the cell migration results are given as area healed, n.s. denotes no statistical significance (*p* > 0.05) at Mann-Whitney test. Data were acquired from 4 independent cell preparations, n ≥ 11 optical fields were evaluated

As previously discussed, the actin cytoskeleton and the ER are uniquely interconnected and dependent on each other for correct function. Hence, any change in ER distribution and structure should be accompanied by a change in the cytoskeleton. Thus, the distribution of the major cytoskeletal protein actin was investigated (Fig. 3A). Incubation with BFA and TG decreased nuclear as well as cytoplasmic actin signal significantly (Fig. 3B). The addition of CytD modified the intracellular distribution of actin (Fig. 3A, B), detectable as a significant increase of the phalloidin fluorescent signal. As observed for the nuclear morphology, disorganization of the cytoskeleton with CytD possibly overruled the response of BFA and TG, in this case the morphometric signatures were clearly dominated by the effect of CytD. In order to verify if the modifications of actin triggered by BFA and TG translate into a functional change of T24 motility, cell migration assays were performed (Fig. 3C). In this context, the collective cell migratory capacity of T24 cells remained unaltered (Fig. 3D).

### Effects of ER stress on cell biomechanics

**Fig. 4:**
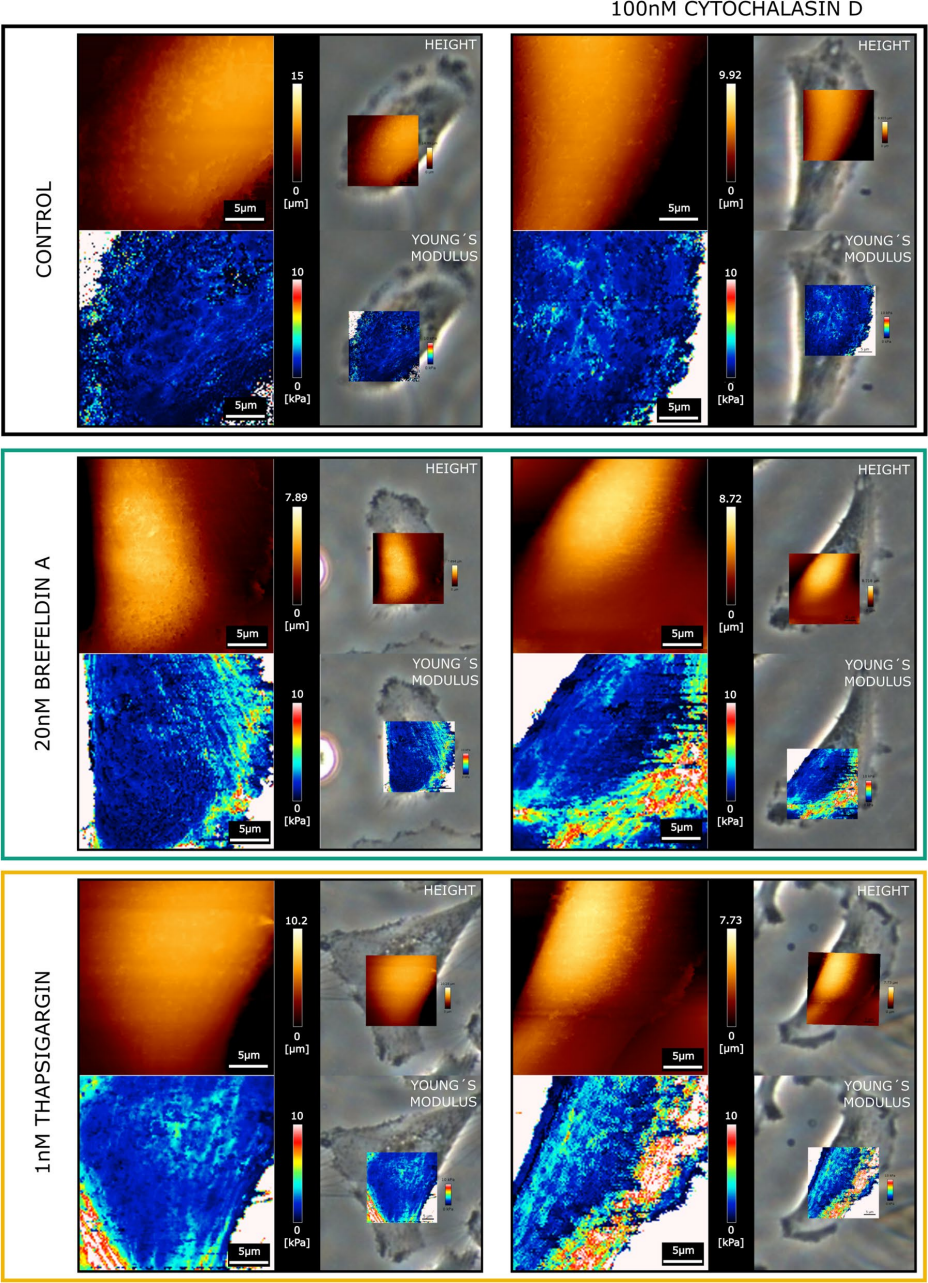
Representative AFM maps of T24 cells treated with 20nM BFA or 1nM TG, with and without 100nM CytD, the top left image of each panel shows the height (orange), the bottom left image the Young´s modulus (maps depicting values between 0–10 kPa). Images on the right side of the panels show the overlay with the corresponding phase contrast images used to identify the different regions of the cell

**Fig. 5:**
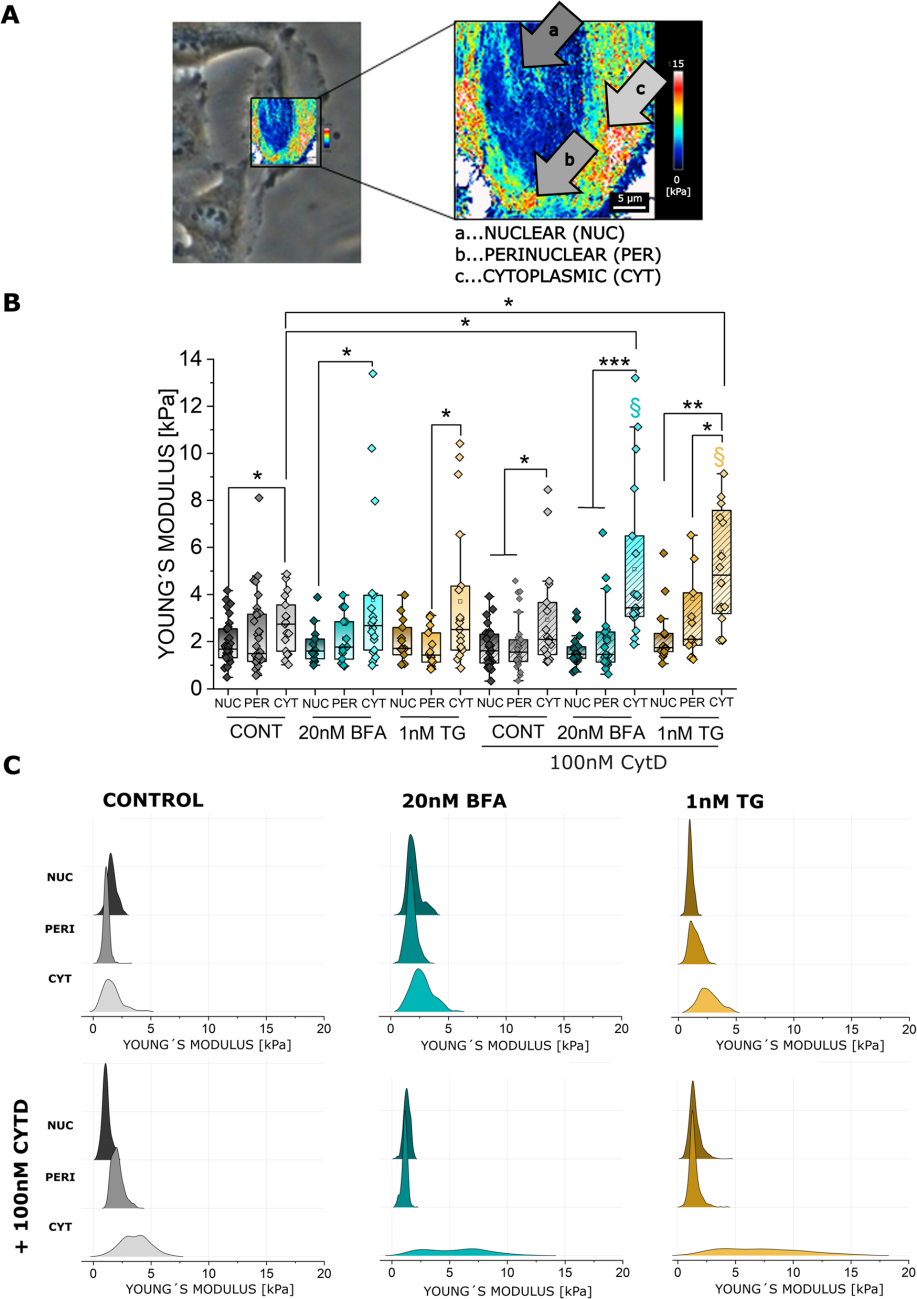
**A** Measurement of the Young´s modulus (YM) at the nuclear (a), perinuclear (b) and cytoplasmic region (c). **B** Quantification of the YM [kPa] per ROI of n ≥ 13 cells per condition. Taken from n ≥ 3 cell preparations (biological replicates). Nuclear (a, NUC), perinuclear (b, PERI) and cytoplasmic (c, CYT) areas were evaluated. Statistical significance is show at Mann-Whitney test, */§ indicates significant difference between treatments (*/§: *p* ≤ 0.05; **: *p* ≤ 0.01; ***: *p* ≤ 0.001). **C** Representative distribution of the Young´s modulus curves in 3.1 µm × 3.1 µm square ROIs, selected for each treatment and cell region (nuclear, perinuclear and cytoplasmic)

Since the ER takes up a significant portion of the cell mass and volume, we started to investigate if the ER rearrangements could be related to modification of the biomechanical properties of the cells. For this, AFM experiments were performed measuring topology and Young´s modulus of T24 cells (Figs. 4 and 5). Measurements were performed taking the morphology of the cells as reference, hence it was possible to correlate the AFM maps with optical images to accurately locate the areas of interest. With this approach, nuclear, perinuclear, and cytoplasmic regions (Fig. 5A) were identified as cell portions that most likely could be affected by ER modulation. In agreement with the lack of toxicity (Fig. 1), no major effects were observed in the morphology of treated cells in comparison to controls (Fig. 4). In this regard, quantification of nuclear stiffness remained constant across the treatments. The most significant changes in cellular stiffness were detected toward the cell periphery, particularly increasing in the cytoplasmic areas. This behavior was described for the controls as well as for the BFA and TG treated cells. Application of CytD returned no variation of this pattern in the control cells. However, actin depolymerization in presence of BFA or TG significantly increased the stiffness in the cell periphery (Fig. 5B, C), retracing the observed redistribution of the ER (Fig. 1E-G).

### PI3K inhibition induced ER rearrangement and effects on cell biomechanics

**Fig. 6:**
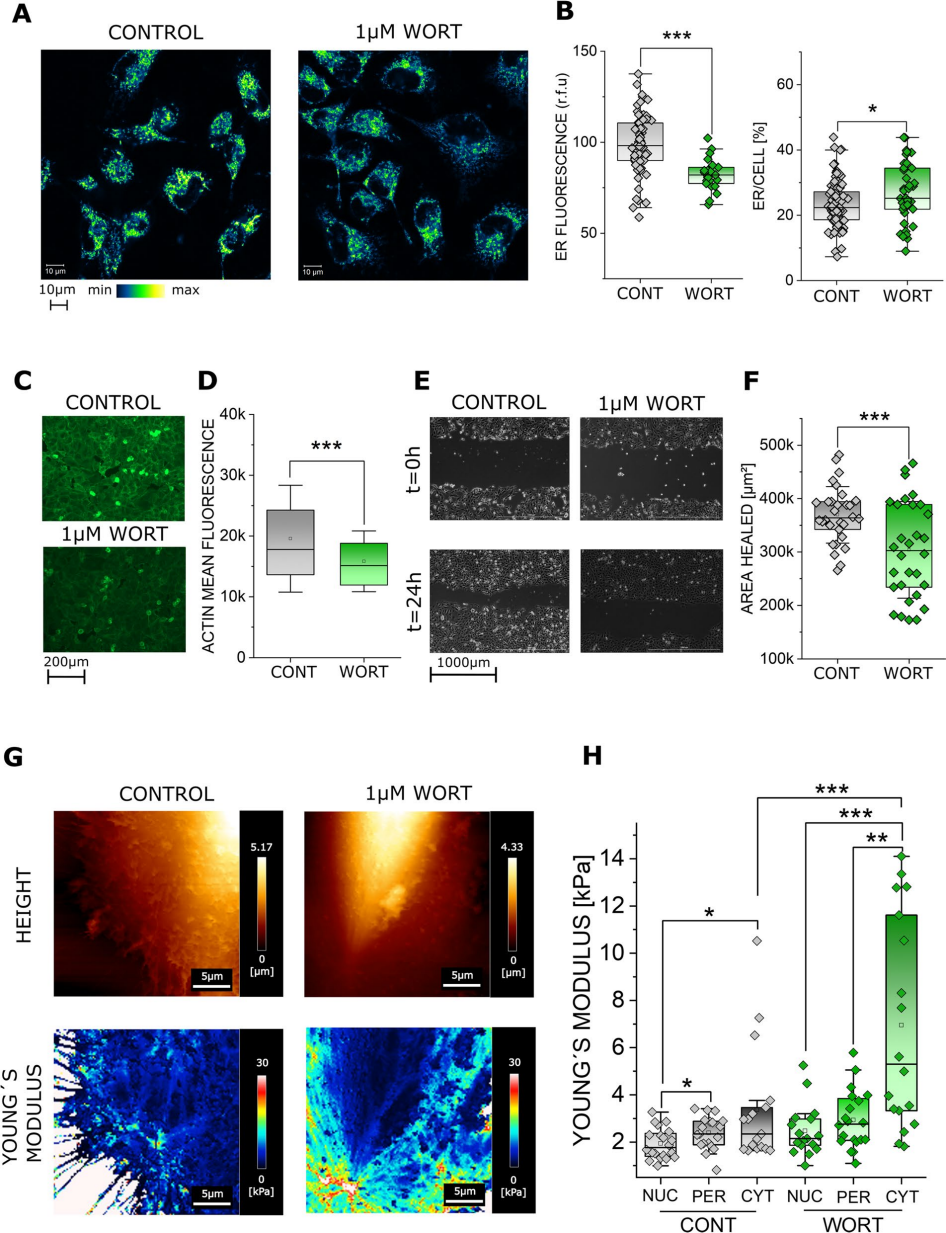
**A** Representative images of T24 cells controls and 24 h incubation with 1µM wortmannin (WORT), ER is shown in blue to yellow. **B** Quantification of the ER staining, results are given as mean fluorescence (r.f.u.) and as the fraction of the cell area covered by the ER signal. * indicates statistical significance at Student´s t-test (*: *p* < 0.05; ***: *p* < 0.001). Results were taken from 3 independent cell preparations and n ≥ 23 ROIs were evaluated. **C** Representative images of actin (green), for control and 1µM wortmannin treated cells. **D** Quantification of the actin signal per cell cytoplasmic area. * Indicates statistical significance at Student´s t-test (***: *p* < 0.001), taken from 3 independent cell preparations across 27 optical fields. **E** Representative images of the gap closure assay, control and 1µM wortmannin treated cells. **F** Quantification of the gap closure assay, results are given as mean area healed over 24 h, * indicates statistical significance at Mann-Whitney test (***: *p* < 0.001). Data were acquired from n ≥ 3 independent cell preparations, n ≥ 10 optical fields were evaluated. **G** Representative AFM height and Young´s modulus [YM] maps of T24 cells controls and 24 h incubation with 1µM wortmannin. **H** Quantification of the YM [kPa] median per ROI for nuclear (NUC), perinuclear (PER) and cytoplasmic (CYT) areas of the cell, n ≥ 17 ROIs were evaluated across n ≥ 3 independent cell preparations. * Indicates statistical significance at Mann-Whitney test (*: *p* < 0.05, **: *p* < 0.01, ***: *p* < 0.001)

In order to further underpin the correlation between ER rearrangement and cell stiffness variation, additional experiments were performed using wortmannin (WORT). WORT is a known inhibitor of PI3K [55] and the PI3K/Akt pathway can be related to the ER stress [56] and unfolded protein response [57]. Incubation with WORT resulted in the rearrangement of the ER, returning a spread of the organelle toward cell periphery possibly without its enlargement (no fluorescent intensity increase; Fig. 6A, B). This was accompanied by a reduction of actin signal intensity (Fig. 6C, D) and by an impairment of cell motility (Fig. 6E, F). Together with WORT induced ER redistribution, a significant increase of the cell stiffness / Young’s modulus was measurable in the cytoplasmic region (Fig. 6G, H).

### Effects of short-term ER stress on T24 cancer cells and normal bladder fibroblasts on cell biomechanics

**Fig. 7:**
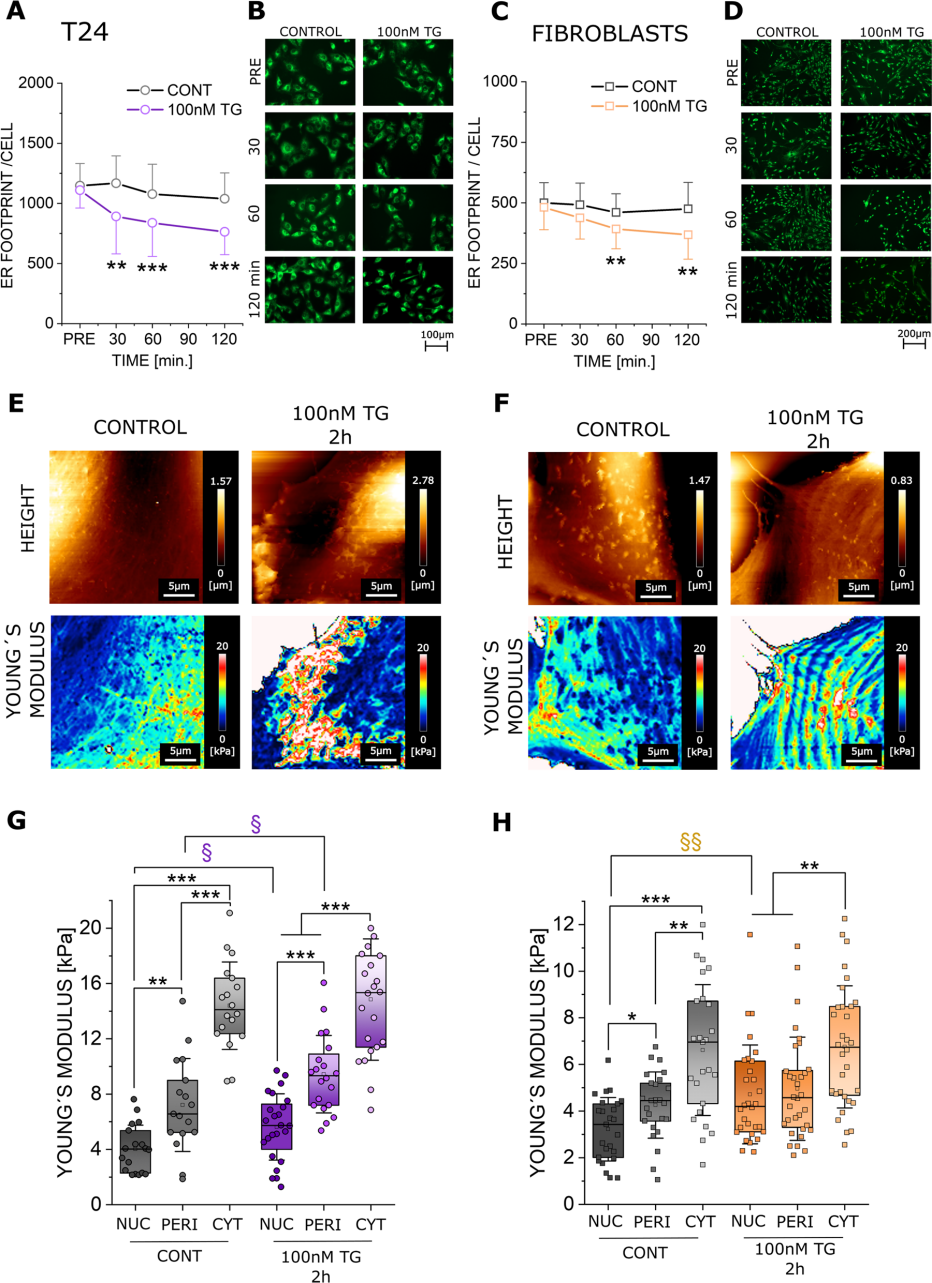
Response of T24 and fibroblasts to short term (1-2 h) incubation with 100nM TG. Quantification of the ER appearance for T24 cancer cells (**A**) and fibroblasts (**C**) respectively, results are given as mean ER footprint area per cell [µm^2^] (average per optical field). * Indicates statistical significance at Mann–Whitney test (n.s. *p* > 0.05; **: *p* < 0.01; ***: *p* < 0.001). Results were taken from 3 independent cell preparations and n ≥ 16 optical fields were evaluated. Representative images of T24 cells (**B**) and fibroblasts (**D**) before treatment (PRE) and after 30, 60, and 120 min incubation with 100nM TG. ER is depicted in green. Representative AFM height and Young´s modulus maps of controls and 100nM TG treated cells (1-2h), T24 (**E**) and fibroblasts (**F**). Quantification of the Young´s modulus per cell in the nuclear (NUC), perinuclear (PERI) and cytoplasmic (CYT) area, for both T24 cells (**G**) and fibroblasts (**H**). */§ Indicates statistical significance at Mann–Whitney test (*/§: *p* < 0.05; **/§§: *p* < 0.01; ***: *p* < 0.001) § indicates differences between controls and cells treated with TG 100nM, * indicates any remaining significances. Results are taken from 3 independent cell preparations from n ≥ 17 cells

Since TG is known to induce also a fast response of the ER [58, 59], this aspect was investigated using a higher concentration (100nM) and shorter incubation time (2 h). Furthermore, to start exploring the validity of these mechanisms also in other cell types, experiments were performed in parallel using normal bladder fibroblasts. Incubation with 100nM TG triggered a retrograde rearrangement of the ER toward the nuclear region in both bladder fibroblasts and cancer cells. This resulted in a significant morphometric change starting from 30 to 60 minutes post-application for T24 and fibroblasts respectively (Fig. 7A-D). For both cell types, this effect could be followed up to 2 h post-incubation (Fig. 7A-D). To investigate if the redistribution of the ER translates into a change in cellular biophysical properties, AFM measurements were performed in the time interval corresponding to stable ER rearrangement (between 1–2 h after TG 100nM incubation). In line with ER localization, Young´s modulus varied significantly in both cell types (Fig. 7E, F) and returned an increase in the nuclear region in comparison to the controls (Fig. 7G, H). Additionally, the Young´s modulus of treated T24 was elevated also in the perinuclear region (Fig. 7G).

### Effects of short-term ER stress on nuclear morphology and actin cytoskeleton in T24 cancer cells and normal bladder fibroblasts

**Fig. 8:**
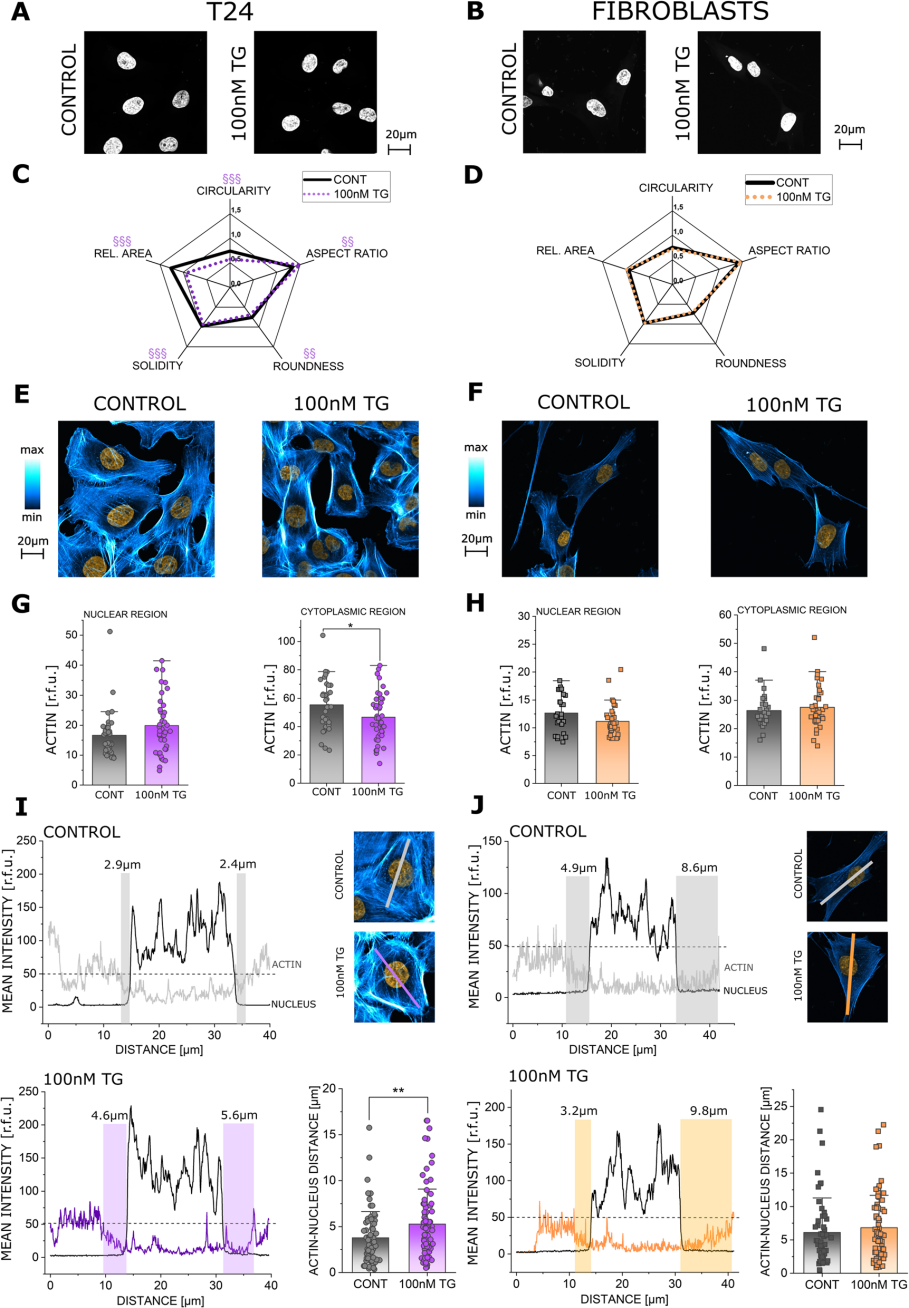
Representative images of DAPI stained nuclei of T24 cells and fibroblasts (**A**, **B**). Nuclear morphology profiles of T24 (**C**) and bladder fibroblasts (**D**), shown are the average values of Rel. Area (Area [µm]/200), roundness, aspect ratio, circularity and solidity (complete list of the average values and standard deviation can be found in supplementary table 1). In total n = 12 optical fields were evaluated, and the average values of all nuclei per image were calculated, for 3 independent cell preparations (biological replicates). Representative images of actin (blue-white) and nuclei (orange) in T24 cells (**E**) and fibroblasts (**F**). Quantification of actin in T24 cells (**G**) and fibroblasts (**H**). N ≥ 36 ROIs for T24 and n ≥ 26 ROIs for fibroblasts were evaluated for each respective subcellular compartment, for 3 independent cell preparations (biological replicates). Quantification and representative images of the distance between actin (phalloidin, blue-white) and nuclear (DAPI, orange) signals for T24 (**I**) and fibroblasts respectively (**J**). Graphical representation of selected cross-sections, showing the signal intensity (r.f.u.) over the distance [µm]; highlighted is the measurement of the gap between the nuclear and actin signals (dotted line marks reference threshold of 50 r.f.u.). In total n ≥ 72 ROIs for T24 and n ≥ 54 for fibroblasts were evaluated, for 3 independent cell preparations (biological replicates). */§ Indicates statistical significance at Student´s t-test (*: *p* < 0.05; **/§§: *p* < 0.01; §§§: *p* < 0.001)

Since nuclear morphology can be tuned in response to mechanical cues and subcellular organization [60, 61], it was postulated that a rearrangement of the ER might correlate to altered nuclear shape. Nuclear morphometric profiling was performed in control cells and cells treated with TG 100nM for 2 h, matching the timeline of the effects on the ER and nuclear stiffness. Here, the nuclei of the T24 cancer cells proved to be uniquely compliant, showing significant changes in all considered descriptors (roundness, area, solidity, circularity and aspect ratio). Application of TG resulted in overall smaller and less uniformly rounded nuclei (Fig. 8A, C). The fibroblasts showed reduced adaption in nuclear morphology (Fig. 8B, D). Additionally, since the nucleoskeleton rearrangement is highly dependent on other cytoskeletal elements [62, 63] and the ER movement would necessarily require also cytoskeletal repositioning, the subcellular distribution of actin was assessed (Fig. 8E–H). Actin signal in the nuclear region remained fairly constant in both cell types and only a decrease of the intensity in cytoplasmic area in the T24 cells could be detected (Fig. 8G, H). Along this line, in T24 cells application of TG (100nM) returned a significant displacement of actin from the perinuclear region (Fig. 8I), which would be compatible with an increased accommodation of the ER. In agreement with the constant morphology of the nucleus, these responses could not be observed for the fibroblasts (Fig. 8J).

## Discussion

Cell biophysical properties are defined by a complex interplay of individual components. Understanding the underlying intricacies is vital, since the resulting parameters such as cell stiffness are emerging players in multiple pathologies [64]. This includes several examples like for instance cancer progression [65, 66], cardiomyopathies and musculoskeletal disorders [67–69], as well as aging [70, 71]. Along this line, the role of cell biomechanical compliance in pathophysiological context is increasingly acknowledged [72–75]. For example, cisplatin resistant ovarian cancer cell lines showed a higher stiffness compared to their nonresistant counterparts, which was accompanied by a dense cytoskeleton of actin stress fibers [76]. Furthermore, via atomic force microscopy (AFM) it is possible to distinguish cancer from non-cancer cells based on their stiffness [77–80] or morphology [81], as well as predict their metastatic potential [82]. Cellular stiffness as a mechanical biomarker is often explained through the actin cytoskeleton [83, 84], i.e. stress fibers and ECM interactions by integrins and focal adhesions. However, cells rely on a broad and sophisticated mechanosensory apparatus [31], which can be even further regulated in the pathological progression. For example, softening of human bladder cancer cells is an important factor in their malignancy, enabling the invasion through tightly packed tissues [83, 85], while cell stiffening was shown to enhance T-cell immunotherapy [86]. In this complex landscape, the role of interwoven cytoskeletal and non-cytoskeletal organelles is difficult to separate. Owing to its dimension, the nucleus contributes substantially to the overall cellular architecture and morphology. Firmly set in a network of actin fibers, the nucleus is connected to the extracellular environment thanks to a continuum system starting from the focal adhesions and binding the nuclear envelope through the LINC complex (linker of nucleoskeleton and cytoskeleton) [87, 88]. From this privileged position, the nucleus acts as a crucial mechanosensor [89, 90], and alteration of its adaptive capacity is related to cellular senescence [91], inflammation [92], or even to failures in the functionality of organs, like in the case of cardiomyopathies [93, 94]. Furthermore, changes in nuclear morphology are connected to cell differentiation, development, and cancer [95–98]. As a matter of fact, the nucleus is not only connected to the cytoskeletal meshwork, but also surrounded by the nuclear envelope, which is in direct contact with the ER. The nuclear envelope can also be thought of as a specialized part of the ER and is even absorbed during mitosis, only to be reformed from the ER´s membrane at later stages [5, 99]. Of similar magnitude is the connection between the ER and the cytoskeleton; the ER moves along microtubules via kinesin mediated sliding, or it is attached to newly forming microtubules via TAC (tip attachment complex) [53]. In turn, ER dynamics can influence microtubules distribution as well [100]. Hence, placed between the nucleus and the cytoskeleton, the ER promises to be a key factor for the regulation of cellular mechanobiology. In the present study, the bladder cancer cell line T24 was chosen as a model based on the unique environment occurring in this tissue in vivo, where cells face a combination of both chemical and physical stressors. On the one side, xenobiotics in urine [101, 102] can act as potential sources of ER stress [103], but also chemotherapeutic drugs and resistance pathways can rely on this mechanism of action [104, 105]. Additionally, physical stress demands biophysical competence and contributes to determine the shape and function of the epithelium [106–109]. In order to investigate the role of the ER in the complex network defining the adaptive biophysical capabilities of bladder cancer cells, two established inducers of ER stress, brefeldin A (BFA) and thapsigargin (TG) were used as model substances levering two distinct pathways [26, 32]. After initial concentration range finding studies (Fig. 1A, B), the effect of the two molecules on the ER was confirmed in absence of significant cytotoxic effects. ER stress manifested as a rearrangement of the ER fluorescent signal across the cell area (Fig. 1E-G), which reflects typical changes in ER morphology, such as membrane expansion, that are a known part of the ER stress response [110, 111]. Coherent variation of the transcription factor CHOP (TG 1nM, Fig. 1C-D) [112] was also observed. Combined modulation of the cytoskeleton via cytochalasin D (CytD) resulted in an enhanced effect for TG but not BFA (Fig. 1E-G). This might be related to known capacity of BFA to alter the cytoskeleton [113], where the addition of CytD would only retrace this response without a noticeable additive effect. However, cell morphology and actin quantification (Fig. 3) do not support a difference in the response of T24 cells to TG and BFA in this regard. Pertaining actin evaluation, it is worth mentioning that this returned a decrease in the signal upon incubation with TG and BFA in comparison to controls (Fig. 3). Indeed, both compounds can alter the actin cytoskeleton [27, 29]. Additionally, this is also compatible with an enlargement of the ER and consequent re-localization of the surrounding cytoskeleton. In this respect, incubation with BFA returned a broader spread of the ER surface in comparison to TG (Fig. 1G). TG, in turn, more efficiently modified nuclear morphology (Fig. 2C-E). Indeed, the ER is closely connected to the nucleus, for example the ER protein REEP3/4 ensures proper architecture of the nuclear envelope [114], therefore changes can mutually reflect on the two components. Furthermore, these changes might be attributed to the connection between the ER and the nucleus through the nuclear envelope [115], as well as to the degradation of the stress fibers stabilizing the nucleus [116]. Results obtained for the ER-cytoskeletal rearrangement in T24 cells would also be in line with the putative mechanism of action of the two compounds; namely, with BFA hampering ER-Golgi vesicle transport [32], possibly in a more peripheric region, in comparison to the action of TG localized to the SERCA [117]. Upon treatment with the ER stressors, cells displayed a rearrangement of the actin cytoskeleton, yet without significant impairment of cell motility (Fig. 3). This on the one side supports the lack of toxicity of the two compounds in the selected experimental concentrations, but also infers for more subtle effects, possibly sustaining the onset of chronic loss of function. Looking for parameters defining the cell’s biomechanics, AFM microscopy is broadly acknowledged as a valuable tool to map cell stiffness [31] and its heterogeneity [80]. The Young´s modulus is a commonly used parameter to describe cell elasticity [118–120]. Indeed, cellular stiffness is not homogeneously distributed across the cell and measurement in a single point, i.e. center of the cell, will not exhaustively represent the complete biophysical profile [77, 121]. Since isolating biophysical properties of different areas is intrinsically related to the scope of the present study, ROIs were selected through direct correlation with optical microscopy (Fig. 5). As previously shown for other cell types [122, 123], stiffness varied in the different intracellular compartments in T24 cells (Fig. 5), returning average values in the nuclear region between 1 and 4 kPa, as previously described [85]. Regardless of the integrity of the actin cytoskeleton (Fig. 3A, B) 24 h treatment with BFA, TG or CytD did not significantly change the Young´s modulus (Fig. 5B, C). Only when the ER was released from the boundaries of the cytoskeleton (CytD treatment, Fig. 5B, C) and the cells were treated with either TG or BFA the stiffness in cytoplasmic compartment increased significantly (24 h, Fig. 5B, C). Even though the contributions of other cytoskeletal elements cannot be excluded at this point, this response mirrored the redistribution of the ER toward the cell periphery as observed in response to ER stress in combination with a disruption of actin polymerization. This seems to support the view that the cytoplasmic “scaffolding” of the ER via actin fibers must be an important factor in the stability and overall cell biomechanics. CytD was previously shown to rather reduce cell stiffness in multiple cell types [85, 124] and it seems unlikely that the morphologic loss of actin cytoskeleton could be the main source of the measured increase in cytoplasmic stiffness. Along this line, Young´s modulus remained constant in control cells even upon application of CytD. In order to investigate whether this effect can be recreated with other molecules affecting ER-cytoskeletal organization*,* experiments were repeated with the PI3K inhibitor wortmannin (WORT), as the PI3K/Akt pathway is crucial in the maintenance of actin integrity [125, 126]. In T24 cells WORT treatment resulted in significant cytoplasmic spread of the ER network (Fig. 6A, B), possibly related to the loss of actin cytoskeletal integrity (Fig. 6C, D) and accompanied by a parallel increase in peripheral stiffness (Fig. 6G, H). Comparing the effects of WORT and BFA, and TG is it worth mentioning that WORT-induced ER-cytoskeletal rearrangement was accompanied by increased intracellular stiffness and loss of cell motility. For TG and BFA, increased stiffness exhibited exclusively in the presence of CytD, which would be in line with the maintained motility described in Fig. 3C, D when the compounds were incubated alone. In order to broaden our investigation on the role of ER rearrangement and cell biomechanical compliance, short term events were also investigated. Indeed, rapid responses are less likely affected by long term contributions as those derived from protein biosynthesis, autophagy or metabolic adaption [112]. Aligning with a time dependent rearrangement of the ER (Fig. 7A-D), in T24 cells TG incubation increased cellular stiffness primarily in the nuclear and perinuclear areas (Fig. 7E and G). Parallel assessment of the actin cytoskeleton revealed a relocation of the actin fibers towards the cell periphery and decreased compactness of the network in the perinuclear region (Fig. 8), which would be compatible with the retrograde accumulation of the ER around the nucleus. In order to start exploring if these pathophysiological changes could be of relevance also for other, non-transformed cell types, parallel experiments were performed on normal bladder fibroblasts. Even if the measured effects were reduced in magnitude in comparison to the cancer cells, also in this case application of TG returned a significant rearrangement of the ER accompanied by an increase of the Young’s modulus in the nuclear region (Fig. 7). Even though further studies are necessary to explore the molecular details of these events, a more modest effect on cell stiffness in comparison to the T24 aligned with a scarce alteration in the actin network and in the underlying nuclear morphology (Fig. 8A-D). In sum, it appears that the ER and the actin cytoskeleton are mutually influencing each other and repositioning during the ER stress response in bladder cells. In this regard, a potential contribution of other cytoskeletal elements or post-translational protein modifications affecting cell morphology cannot be ruled out and remains an open and intriguing research question. Nevertheless, the variation of cell stiffness measured via AFM (Figs. 5, 6 and 7) largely aligned with the subcellular localization of the ER (Figs. 1, 6 and 7) and scarcely with the actin cytoskeleton (Figs. 2, 6 and 8) allowing to postulate a contribution for the ER in the maintenance of cell biomechanics.

## Conclusion

The present study supports the importance of the ER-cytoskeleton network, in maintaining cell biophysical properties in relation to ER stress response. This opens the possibility of a novel role for the ER in supporting cell structure. Here, the ER is suggested to be an important contributor to cellular stiffness in bladder cells, working coherently with both the cyto- and nucleoskeleton to stabilize overall cell architecture and nuclear morphology. Pharmacological manipulation of the individual components of this complex network resulted in significant changes in cell mechanobiology. These results promise to be relevant not only for bladder cancer biophysical adaptiveness and aggressiveness, but also for the comprehension of chronic pathophysiological processes related to ER dysfunction and for the identification of new druggable targets and therapeutic interventions.

### Supplementary Information

**Additional file 1:** **Supplementary table 1.** Average values and standard deviations of nuclear morphology, corresponding to the panels Fig. 8C and D.

## Data Availability

The data that support the findings of this study are available from Giorgia Del Favero, [GDF], upon reasonable request.
