# Supplementary material for: Intracellular remodeling associated with endoplasmic reticulum stress modifies biomechanical compliance of bladder cells
Source: Cell Commun Signal. 2023 Oct 30;21:307. doi: 10.1186/s12964-023-01295-x (PMC10614373; doi:10.1186/s12964-023-01295-x)
Supplement: Supplementary file 2 — Additional file 1: Supplementary table 1. Average values and standard deviations of nuclear morphology, corresponding to the panels Fig. 8C and D. [file 12964_2023_1295_MOESM1_ESM.docx]

Intracellular remodeling associated with endoplasmic reticulum stress modifies biomechanical compliance of bladder cells

Livia Gruber (LG) ^1+^, Maximilian Jobst (MJ) ^1,2,3+^, Endre Kiss (EK) ^2^, Martina Karasová (MK) ^1,2^, Bernhard Englinger (BE) ^4,5^, Walter Berger (WB) ^5^, Giorgia Del Favero (GDF) ^1,2^*

1 Department of Food Chemistry and Toxicology, University of Vienna Faculty of Chemistry, Währingerstr. 38-40, 1090 Vienna, Austria.

2 Core Facility Multimodal, Imaging, University of Vienna Faculty of Chemistry, Währingerstr. 38-40, 1090 Vienna, Austria.

3 University of Vienna, Vienna Doctoral School in Chemistry (DoSChem), Währinger Str. 42, 1090 Vienna, Austria.

4 Department of Urology, Comprehensive Cancer Center, Medical University of Vienna, 1090, Vienna, Austria.

5 Center for Cancer Research and Comprehensive Cancer Center, Medical University Vienna, 1090, Vienna, Austria.

+ These authors contributed equally to this work (Shared first authorship)

* Correspondence: G. Del Favero, giorgia.del.favero@univie.ac.at

*Supplementary table 1: Average values (AVG) and standard deviations (STDDEV) of nuclear morphology (circularity (CIRC), roundness (ROUND), solidity (SOL) aspect ratio (AR), area and relative area (REL. AREA)), for T24 cells treated for 2 h with 100 nM thapsigargin (TG), corresponding to the panels Figure 8 C and D.*

|  |  |  | CIRC | ROUND | SOL | AR | AREA [µm²] | REL. AREA |
| --- | --- | --- | --- | --- | --- | --- | --- | --- |
| **T24** | CONT | AVG | **0,74** | **0,76** | **0,98** | **1,35** | **253,85** | **1,27** |
|  |  | STDDEV | 0,06 | 0,05 | 0,01 | 0,11 | 29,45 | 0,15 |
|  | 100nM TG 2h | AVG | **0,57** | **0,69** | **0,93** | **1,50** | **189,99** | **0,95** |
|  |  | STDDEV | 0,10 | 0,05 | 0,03 | 0,10 | 24,67 | 0,12 |
| **FIBROBLASTS** | CONT | AVG | 0,76 | 0,72 | 0,98 | 1,43 | 186,83 | 0,93 |
|  |  | STDDEV | 0,07 | 0,11 | 0,02 | 0,22 | 35,02 | 0,18 |
|  | 100nM TG 2h | AVG | 0,72 | 0,70 | 0,97 | 1,48 | 196,49 | 0,98 |
|  |  | STDDEV | 0,09 | 0,09 | 0,01 | 0,19 | 41,25 | 0,21 |
